# Early cardiac rehabilitation in ICU for infants after complex congenital heart disease surgery: a retrospective case series

**DOI:** 10.3389/fped.2026.1790456

**Published:** 2026-05-04

**Authors:** Mingye Yue, Hongjun Deng, Jiawei Shi, Yuting Hu, Xinghong Liu, Huihua Wang

**Affiliations:** Union Hospital, Tongji Medical College, Huazhong University of Science and Technology, Wuhan, Hubei, China

**Keywords:** cardiac rehabilitation, congenital heart disease, early rehabilitation, infant, intensive care unit

## Abstract

**Background:**

Complex congenital heart disease (CHD) has long been a significant cause of infant mortality and severe morbidity. However, pediatric cardiac rehabilitation (CR) is gaining recognition, and evidence regarding postoperative ICU phase CR for infants with CHD remains scarce.

**Methods:**

This was a retrospective case series, we reviewed clinical data for 10 infants with complex CHD who received an early, individualized CR program during the postoperative ICU phase. The rehabilitation was initiated between postoperative days 5–15 (median 9). The program was based on an exercise prescription, and integrated multidimensional interventions, including respiratory training, gross motor function training, nutritional support, and developmental care.

**Results:**

All infants were critically ill postoperatively requiring various forms of life-support therapy in the ICU. No serious adverse events related to rehabilitation occurred; transient fluctuations in vital signs resolved promptly with temporary pauses in therapy. Eight infants were discharged home after recovery, and two were transferred to other institutions. Functional improvements were observed across respiratory, feeding, neuromotor, and circulatory domains during the ICU stay.

**Conclusion:**

For infants with complex CHD, early initiation of a cardiac rehabilitation program during the ICU phase appears safe and feasible when implemented under multidisciplinary assessment and close clinical monitoring. This approach may support functional recovery across cardiopulmonary and neuromotor domains during a critical developmental window.

## 1 Introduction

Congenital heart disease (CHD) is one of the most common birth defects, with complex forms representing a major cause of infant morbidity and mortality (1). Although advances in surgical techniques and perioperative care have substantially improved early survival rates for infants undergoing complex CHD surgery, anatomical correction does not guarantee favorable functional or neurodevelopmental outcomes. Approximately 25%–50% of these infants experience delayed cardiopulmonary recovery, neurodevelopmental impairments, growth retardation or motor delays, all of which can adversely affect long-term quality of life (2–5).

Cardiac rehabilitation (CR), traditionally defined as a multidisciplinary, exercise-centered intervention, has been widely validated in adults and older children with heart disease, demonstrating benefits for functional status, exercise tolerance, and long-term prognosis (6–11). However, existing pediatric CR research has primarily focused on stable children over three years of age (7, 12). Infants represent a unique and clinically important population, as many complex CHD surgeries are performed during early infancy. In addition, infancy is a critical period for neurodevelopment and motor maturation, during which early activity-based interventions may help to optimize long-term developmental outcomes. Consequently, evidence regarding CR in infants—particularly during the intensive care unit (ICU) recovery phase after complex CHD surgery—remains scarce. This postoperative period represents a vulnerable yet critical window for cardiopulmonary, neuromotor, and developmental recovery. However, clinical instability and the need for life support pose significant challenges to implementing rehabilitation, leaving practical experience and safety data in this population notably limited.

To address this gap, we conducted a retrospective review of early individualized CR practices in the ICU for 10 infants following complex CHD surgery at our institution, with a focus on feasibility, safety and implementation characteristics. This study aims to provide preliminary evidence to support the clinical application and future standardization of early CR for infants with complex CHD.

## 2 Methods

### 2.1 Study design and setting

This retrospective case series was conducted at a tertiary general hospital. We reviewed the demographic and perioperative clinical data of infants with complex CHD who underwent biventricular repair surgery and subsequently received early CR interventions in the ICU. The primary aims of this study were to describe the implementation characteristics of an early CR program and to assess its feasibility and safety in this postoperative infant population. The study was approved by the Ethics Committee of Union Hospital, Tongji Medical College, Huazhong University of Science and Technology. The need for individual consent was waived by the committee.

### 2.2 Participants and data collection

We retrospectively reviewed the clinical data of 10 infants with complex CHD who underwent definitive biventricular repair surgery and received early postoperative CR in the ICU in 2025 (Table 1). The cohort comprised 5 males and 5 females. The median age at surgery was 1.9 months (range 17 days–12 months), and the median body weight was 3.8 kg (range 3.1–9.0). All infants had a confirmed preoperative diagnosis of complex CHD based on echocardiography and other relevant examinations. Postoperatively, they were admitted to the ICU for standard critical care management. Early CR interventions were initiated following a multidisciplinary assessment, once relative hemodynamic stability allowed.

**Table 1:** Baseline characteristics, postoperative ICU course, and CR initiation in infants after definitive repair of complex CHD.

Case	Age at surgery	Weight (kg)	Diagnosis	Surgery	Delayed sternal closure	PD	iNO	Reintubation	MV (days)	CR initiation^a^ (POD)	CR duration (days)	Disposition
1	17 d	3.2	TAPVC+PDA	TAPVC Repair+PDA Ligation	Yes	Yes	No	Yes	45.5	15	63	Transfer
2	4 m 17 d	7	TOF+PFO+Bilateral SVC	TOF Repair+PFO Repair	No	No	No	No	10.9	8	22	Discharged
3	1 m 3 d	3.3	CAVSD+PDA	CAVSD Repair+PDA Ligation	No	No	No	Yes	23.6	10	12	Discharged
4	10 m 22 d	7.8	TGA+VSD+PH	ASO+VSD Repair	Yes	Yes	Yes	No	6.5	7	14	Discharged
5	1 m 15 d	3.4	IAA+ASD+VSD+PDA	IAA Repair+ASD Repair+VSD Repair+PDA Ligation	Yes	Yes	No	Yes	17.3	5	25	Discharged
6	2 m 11 d	3.7	TGA+VSD	ASO+VSD Repair	No	No	No	Yes	5.2	10	11	Discharged
7	2 m 9 d	4.3	TGA+VSD	ASO+VSD Repair	No	No	No	Yes	37.6	8	45	Transfer
8	18 d	3.1	TAPVC+PDA+ASD	TAPVC Repair+PDA Ligation+ASD Repair	Yes	No	No	No	5.4	10	6	Discharged
9	1 m	3.9	IAA+VSD+PDA+PFO	IAA Repair+VSD Repair+PDA Ligation+PFO Repair	No	No	No	No	4.7	11	11	Discharged
10	12 m	9	CAVSD+PDA+PH	CAVSD Repair+PDA Ligation	No	No	Yes	No	1.8	5	6	Discharged

CR, cardiac rehabilitation; CHD, congenital heart disease; PD, peritoneal dialysis; iNO, inhaled nitric oxide; MV, mechanical ventilation; POD, postoperative day; TAPVC, total anomalous pulmonary venous connection; PDA, patent ductus arteriosus; TOF, tetralogy of fallot; PFO, patent foramen ovale; SVC, superior vena cava; CAVSD, complete atrioventricular septal defect; TGA, transposition of the great arteries; VSD, ventricular septal defect; PH, pulmonary hypertension; ASO, arterial switch operation; IAA, interrupted aortic arch; ASD, atrial septal defect. Age is presented as months (m) or months/days (m/d) as appropriate.

a CR initiation was defined as the onset of exercise interventions.

### 2.3 Standard postoperative care

At our institution, the standard postoperative management for infants after complex congenital heart surgery comprises life-sustaining critical care focused on physiological stabilization. This includes pharmacologic support, mechanical ventilation, management of organ dysfunction, routine nutritional advancement, and essential nursing care. A structured, multidisciplinary cardiac rehabilitation program was not part of routine care during the ICU stay.

### 2.4 Early cardiac rehabilitation protocol for infants

**Figure 1:**
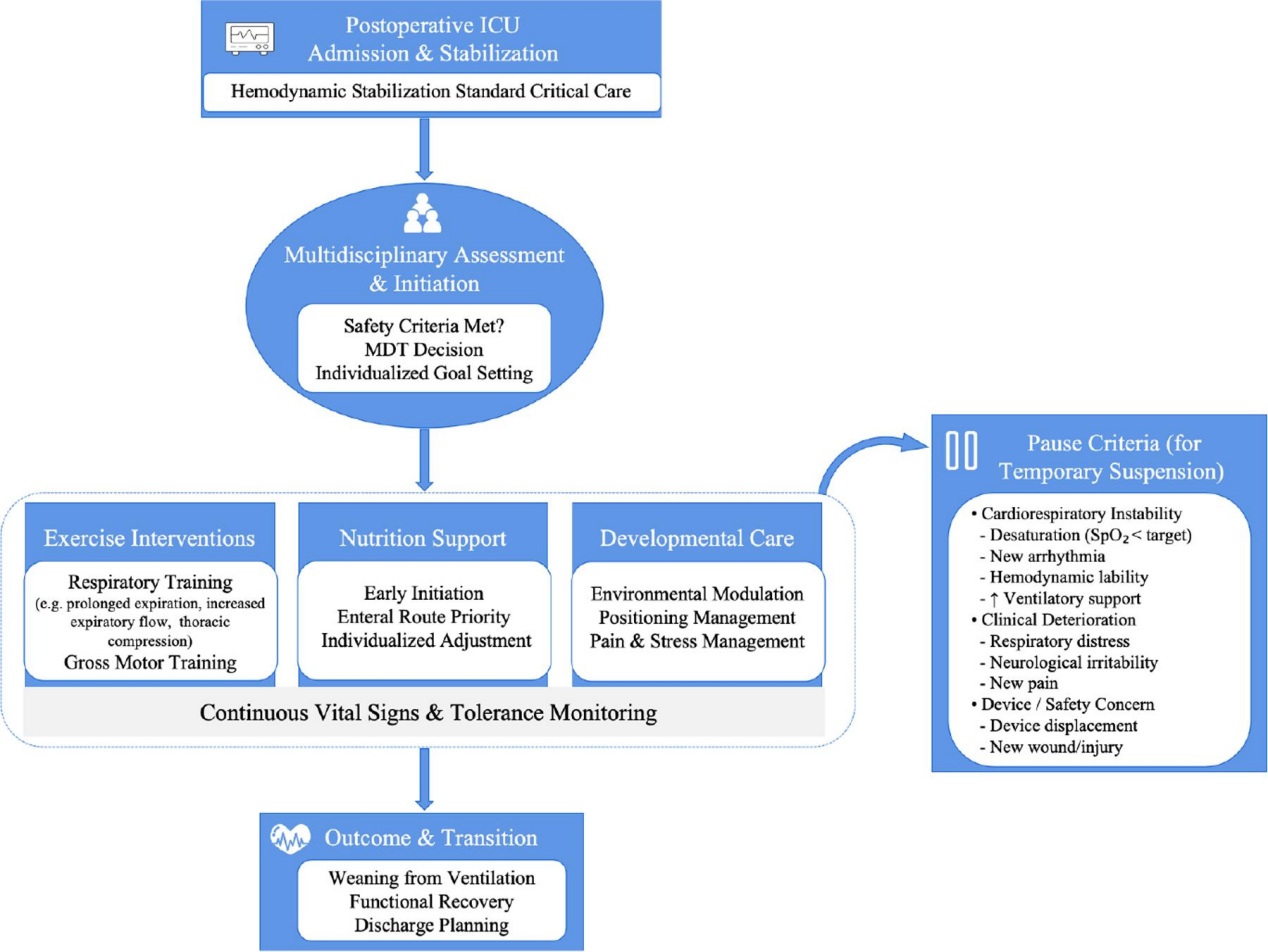
Workflow of the early postoperative cardiac rehabilitation protocol forinfants. Initiation follows multidisciplinary safety assessment. The core exercise prescription is implemented alongside synergistic modules for nutrition and developmental care, with dynamic adjustment based on continuous clinical monitoring.

Infants differ from adults and older children in their cognitive maturation, cardiopulmonary physiology, and motor development. For infants with complex CHD, the postoperative ICU period constitutes a critical window of neuro-motor and cardiopulmonary recovery, during which standard rehabilitation models are not directly applicable. To address these specific developmental and clinical needs, we implemented a structured CR protocol developed at our institution. Grounded in established rehabilitation principles and multidisciplinary collaboration, this protocol emphasized the promotion of motor function while integrating dedicated nutritional support and developmental care. All infants received rehabilitation according to this protocol, with individualized adaptations based on their clinical stability and tolerance. The overall implementation workflow is summarized in Figure 1.

#### 2.4.1 Postoperative rehabilitation assessment

CR was initiated during the postoperative ICU phase, guided by the principles of safety, individualized assessment, and multidisciplinary decision-making. Clinical indications for initiating rehabilitation included impaired motor function, difficult weaning or failure to progress in weaning within the expected postoperative timeframe (often perceived as prolonged mechanical ventilation), hypercapnia (CO₂ retention), abnormal respiratory rate, right heart dysfunction, impaired neurobehavioral responsiveness, and feeding difficulties.

Prior to initiation, a joint assessment was conducted by a multidisciplinary team. The ICU physician was responsible for overall intensive care management and stability assessment; the cardiac surgeon evaluated surgical outcomes and cardiac function; the clinical dietitian performed nutritional risk screening and developed support plans; the rehabilitation therapist conducted respiratory and motor interventions; and the ICU nursing staff assisted with postural management, respiratory training, nutrition support, and developmental care. Coordination was achieved through multidisciplinary rounds where the infant's progress, tolerance, and rehabilitation goals were discussed. The rehabilitation plan was documented in the medical record and updated based on these discussions and the infant's condition.

**Absolute contraindications** to initiating rehabilitation included: (1) Acute postoperative complications present at or arising after ICU admission, such as delayed sternal closure, unstable fractures, intracranial hemorrhage, uncontrolled active bleeding, or severe postoperative infection (e.g., sepsis or septic shock); (2) Airway conditions requiring surgical intervention or acute respiratory complications; (3) Severe arrhythmias, including frequent ventricular premature contractions at rest or with activity, short runs of ventricular tachycardia, or advanced atrioventricular block; (4) Ischemic ST-T changes at rest or with minimal activity; (5) Severe heart failure; (6) Postoperative pulmonary hypertensive crisis; (7) Postoperative low cardiac output syndrome.

**Criteria for pausing rehabilitation** (temporary suspension) were: (1) Cardiopulmonary instability, defined as an increase in the FiO₂ requirement by ≥20% from baseline (or an absolute FiO₂ > 60%) or a decrease in SaO₂ by ≥10% from the clinical target; (2) New-onset arrhythmias or ST-segment depression >3.0 mm; (3) Dyspnea, tachypnea, compromised airway integrity, increased ventilatory support requirements, or patient-ventilator asynchrony; (4) A decrease in systolic or diastolic blood pressure >20% from baseline, or a pulse pressure <20 mmHg; (5) Altered mental status or new neurological irritability; (6) New wounds or musculoskeletal injuries; (7) Displacement of airway or vascular access devices; (8) Symptoms such as significant fatigue, restlessness, or cyanosis; (9) Any incident with a risk of injury (e.g., a fall from bed). Rehabilitation was resumed only after clinical stability was restored and a multidisciplinary consensus was reached.

#### 2.4.2 Exercise interventions

Interventions were initiated 5–15 days postoperatively, with 2–3 daily sessions of 15–30 min each, and were dynamically adjusted according to each infant's clinical tolerance. The exercise intervention which included respiratory rehabilitation and gross motor training, aimed to improve postoperative respiratory function, promote motor recovery, and support neuromotor development. During mechanical ventilation, bedside passive interventions were primarily used, with duration and intensity gradually increased after extubation. All procedures were performed by a dedicated team of rehabilitation therapists trained in pediatric cardiac rehabilitation, with ICU nursing staff assisting in postural management, respiratory training, and other supportive tasks.

##### 2.4.2.1 Respiratory rehabilitation

1. **Respiratory Training.** In infants, the goal of respiratory training is not to enhance muscle strength—as is typical in adults—but to maintain airway patency, promote lung expansion, optimize gas exchange, and prevent pulmonary complications. Due to their immature cognitive development, infants cannot cooperate with active breathing exercises; their respiratory system compensates for stress primarily through tachypnea rather than by increasing tidal volume. Therefore, our respiratory training emphasized passive techniques designed to regulate respiratory rate and establish an abdominal breathing pattern. Core techniques included prolonged expiration, increased expiratory flow, and thoracic compression. For mechanically ventilated infants, training was conducted during synchronized or assisted ventilation modes, provided spontaneous respiratory efforts were present. This aimed to support the development of spontaneous ventilation and facilitate weaning. For non-ventilated infants, the focus shifted to improving breathing rhythm and mitigating hypoventilation. In infants with concomitant diaphragmatic dysfunction (Cases 1 and 7), external diaphragm pacing was applied as an adjunctive modality after contraindications were excluded.
2. **Airway Clearance.** Airway clearance interventions facilitated secretion drainage and segmental lung expansion through postural changes, prone positioning, and percussion techniques. For mechanically ventilated infants who met safety criteria, prone positioning was implemented under close monitoring to improve ventilation-perfusion matching and assist with secretion clearance. The decision to use prone positioning was individualized, depending on clinical stability, airway security, and hemodynamic tolerance. Consequently, not all ventilated infants received this intervention. Some infants (Cases 1, 3, and 7) received both prone positioning during mechanical ventilation and awake prone positioning after extubation, while others received only the latter. The duration of prone positioning ranged from ≥12 h per day during mechanical ventilation to 6–8 h per day after weaning, adjusted based on tolerance and clinical status.

##### 2.4.2.2 Gross motor training

Gross motor training aimed to prevent ICU-acquired weakness and promote neuromotor development, guided by individualized and progressive principles. Before intervention, each infant's developmental level was assessed using the Peabody Developmental Motor Scales–2 (PDMS–2). Rehabilitation plans were then formulated to target the promotion of age-appropriate motor milestones, based on the infant's postoperative clinical status and developmental stage.

1. **Individualized Rehabilitation Plan**. Rehabilitation activities included passive joint mobilization, gentle massage, core stabilization exercises, limb muscle activation, and balance training. For infants with abnormal muscle tone or restricted joint range of motion, interventions focused on modulating muscle tone and improving joint mobility. For older infants demonstrating emerging active motor abilities, training incorporated sitting balance practice, prone head lifting, and toy-guided grasping and visual tracking activities to integrate motor and cognitive development.
2. **Dynamic Adjustment.** Rehabilitation for infants is inherently dynamic and must be aligned with the clinical recovery trajectory. For example, Case 6 required reintubation due to postoperative hypercapnia and began rehabilitation on postoperative day 10. During the period of mechanical ventilation, the focus was on passive mobilization and musculoskeletal maintenance to prevent complications and support weaning readiness. Following successful extubation, the emphasis shifted to consolidating respiratory function and encouraging active movement. Positional management, visual tracking, and grasping activities were introduced to promote cognitive-motor integration. As tolerance improved, this progressed to head control and prone balance training. This stepwise approach ensured that rehabilitation milestones were aligned with clinical recovery, facilitating a transition from life-support dependence toward functional advancement.

#### 2.4.3 Nutrition support

The nutrition prescription served as a cornerstone of postoperative rehabilitation for infants with complex CHD. These infants frequently presented with preoperative feeding difficulties and malnutrition secondary to cardiac insufficiency. In the postoperative period, they faced the combined challenges of surgical stress and a hypermetabolic state, making comprehensive nutritional support critical for cardiopulmonary recovery and neuromotor development. Accordingly, we implemented a nutritional management strategy guided by the principles of “early initiation, enteral route priority, and individualized adjustment”.

Nutritional risk was assessed using the STRONGkids screening tool within 24 h of ICU admission. Phase-specific nutritional goals were then established based on the clinical condition: during the acute phase, the focus was on maintaining metabolic homeostasis and preventing further nutritional deterioration; during the stable phase, the goal shifted to promoting catch-up growth. The route of nutritional support was dynamically adjusted according to hemodynamic status and gastrointestinal tolerance. We employed a stepwise transition from parenteral nutrition (PN) to enteral nutrition (EN), utilizing combined PN and EN support when necessary to ensure the delivery of adequate total energy and protein.

Minimal enteral nutrition (trophic feeds) was initiated as early as possible once hemodynamic stability was achieved. Enteral feeding volumes were then gradually advanced while concomitantly reducing and ultimately discontinuing PN, with the goal of achieving full enteral nutrition. For infants exhibiting feeding intolerance, individualized strategies—such as modifying the feeding route (e.g., switching from gastric to post-pyloric), adjusting the infusion rate, or changing the nutritional formula—were employed. Prior to transitioning to oral feeding, a bedside swallowing function assessment was performed. For infants identified with swallowing-breathing coordination disorders or low feeding efficiency, targeted swallowing therapy and feeding guidance were provided.

#### 2.4.4 Developmental care

Developmental care is recognized to confer neurobehavioral and health benefits for infants with complex CHD (13). Its core objective is to proactively modify the ICU environment and care processes to mitigate iatrogenic stress and create a developmentally supportive microenvironment (14). In this study, our developmental care approach focused on three key domains:

1. **Low-Stress ICU Microenvironment.** Continuous alarms, artificial lighting, and frequent care procedures in the ICU can disrupt sensory regulation and sleep-wake cycles. To create a more developmentally appropriate environment, we implemented the following measures: (i) Noise control: reducing monitor alarm volumes, minimizing non-essential conversations, handling equipment gently, and instituting designated “quiet hours” while maintaining safety monitoring; (ii) Light regulation: simulating natural circadian rhythms by using blackout covers at night to promote sleep and maintaining soft, indirect lighting during daytime hours; (iii) Care clustering: bundling routine nursing procedures (e.g., vital sign checks, diaper changes) to minimize sleep disruptions.
2. **Development-Guided Positioning.** Therapeutic positioning supports neuromuscular development and helps prevent complications. Our key strategies included: (i) Prone positioning: implemented in multiple, supervised sessions daily for alert infants with stable vital signs to promote pulmonary expansion, secretion drainage, and early gross motor development (15); (ii) Physiological positioning: in the supine position, using rolled blankets or swaddling to create a contained, “nest-like” boundary. This approach, consistent with developmental care and NIDCAP principles, facilitated a flexed posture with shoulders forward and hips flexed, promoting self-soothing behaviors such as hand-to-mouth exploration.
3. **Systematic Pain and Stress Management.** Pain and stress are significant disruptors of neurobehavioral development. Our management protocol included: (i) Routine assessment: using a validated pediatric pain scale at least every 4 h; (ii) Pre-procedural non-pharmacological interventions: prioritizing techniques such as non-nutritive sucking, facilitated tucking, and skin-to-skin contact (when feasible) prior to painful procedures to attenuate nociceptive responses (16); (iii) Minimal sedation strategy: employing the lowest effective dose of sedatives when medically necessary to mitigate sedation-related neurodevelopmental risks and support opportunities for early interaction. (iv) Coordination with rehabilitation activities: rehabilitation was dynamically coordinated with the clinical state. Sessions were scheduled to avoid periods of significant pain or acute withdrawal distress. For infants on sedative/opioid weaning protocols, therapy was scheduled during periods of optimal alertness and comfort. Close communication between therapists, nurses, and physicians ensured sessions were conducted when the infant was most receptive.

## 3 Results

### 3.1 Patient characteristics and postoperative course

All 10 infants underwent definitive surgical correction of cardiac malformations under general anesthesia with cardiopulmonary bypass. Postoperatively, they were transferred to the ICU for standard critical care, which included circulatory support, mechanical ventilation, antimicrobial therapy, and other organ support as needed. Several infants experienced complications that required additional interventions, including delayed sternal closure for myocardial edema, peritoneal dialysis for acute kidney injury, and inhaled nitric oxide therapy for postoperative pulmonary hypertension. Notably, no infant required mechanical circulatory support (e.g., ECMO or VAD). Overall, the cohort exhibited critical postoperative illness with a high degree of dependence on life-support therapies.

### 3.2 Safety and feasibility of rehabilitation

Individualized CR was initiated between postoperative days 5 and 15 (median 9), concurrent with ongoing critical care. Eight infants were subsequently discharged home following recovery. The remaining two infants were transferred to other institutions for continued care. No serious adverse events directly attributable to the rehabilitation program were observed. Transient decreases in oxygen saturation (typically <5% from baseline) occurred during some therapy sessions; these resolved spontaneously within 5 min of pausing the activity. Rehabilitation sessions were temporarily suspended for several infants due to events such as fever, tachycardia, or clinical instability, and were resumed once these conditions stabilized. Notably, Case 1 continued to participate in the rehabilitation program after undergoing a second surgical procedure. These findings suggest that early, individualized rehabilitation is clinically feasible and appears to have an acceptable safety profile when conducted under close monitoring and with predefined safety criteria.

### 3.3 Functional recovery

#### 3.3.1 Respiratory trajectory

The median duration of mechanical ventilation was 8.7 days (range 1.8–45.5). Five infants were extubated successfully on the first attempt. Three infants required reintubation but were subsequently weaned successfully with the continuation of rehabilitation and standard postoperative management. Progressive improvements in spontaneous respiratory effort, airway clearance, and reductions in oxygen support requirements were observed with clinical stabilization.

#### 3.3.2 Feeding trajectory

EN was initiated early, at a median of postoperative day 1.0 (range 0.7–2.3). PN was required for a median of 1.5 days (range 0–14), with its use progressively tapered as EN was advanced. Exclusive EN was achieved at a median of postoperative day 5 (range 1–23). All infants transitioned to exclusive EN by ICU discharge, reflecting recovery of gastrointestinal function, decreased interference between respiratory effort and feeding, and improved neuromotor responsiveness. Several infants had also initiated oral feeding by the time of transfer from the ICU.

Nutritional status was monitored throughout the ICU stay. The median weight at rehabilitation initiation was 3.8 kg (range 3.1–9.0 kg). At ICU discharge, the median weight was 4.0 kg (range 3.2–9.0 kg), reflecting the minimal weight gain typical during the acute postoperative catabolic phase prior to the convalescent growth period.

#### 3.3.3 Motor developmental trajectory

Neuromotor performance improved gradually in parallel with increasing clinical stability. Several infants achieved age-appropriate milestones such as head control or prone positioning activities. Concurrently, reductions in abnormal muscle tone and an increase in the frequency and quality of spontaneous movements were noted. These improvements became more pronounced after the transition from mechanical ventilation to awake rehabilitation.

#### 3.3.4 Hemodynamic trajectory

Hemodynamic recovery followed a comparable pattern. The median vasoactive-inotropic score decreased from 7.62 prior to rehabilitation initiation to 4.31, indicating a progressive reduction in the need for circulatory support during the ICU stay.

Overall, these functional recovery trajectories did not follow a strictly linear progression and varied among infants, influenced by factors such as postoperative complications, clinical stability, and individual tolerance to rehabilitation. This case series illustrates that postoperative recovery in critically ill infants is a multidimensional process, involving concurrent progress across several functional domains rather than achievement of a single endpoint.

## 4 Discussion

This case series describes the implementation and outcomes of an early, infant-adapted cardiac rehabilitation (CR) protocol during the postoperative ICU stay for infants with complex CHD. Conducted under rigorous multidisciplinary assessment and continuous monitoring, the protocol demonstrated acceptable safety and feasibility, even in infants with diverse critical postoperative conditions requiring mechanical ventilation, peritoneal dialysis, or inhaled nitric oxide therapy. No serious adverse events were attributed to the rehabilitation interventions, and transient vital sign fluctuations resolved promptly upon pausing the activity. These findings corroborate earlier reports (17, 18) and reinforce the view that complex CHD and the need for postoperative life support should not be considered absolute contraindications to early, carefully monitored rehabilitation. More importantly, they directly address the critical evidence gap identified at the outset of this study, by demonstrating that a structured, infant-specific CR program—integrating exercise, nutrition, and developmental care—can be safely and feasibly integrated into the postoperative ICU care of this vulnerable population.

Introducing such structured rehabilitation during this early, critical phase is particularly salient because infants undergoing complex CHD surgery are at high risk for motor delays and neurodevelopmental vulnerability (19). While most pediatric CR research has focused on older, stable children after ICU discharge, the immediate postoperative period in the ICU may represent a critical window of opportunity for intervention. Advancing rehabilitation interventions into this phase may help mitigate the risk of ICU-related motor decline and create a supportive window for functional recovery, consistent with the concept of a critical period for early rehabilitation intervention (20, 21). The variability in rehabilitation initiation timing observed in our cohort (postoperative days 5–15) underscores the necessity for clinical flexibility and individualized judgment when integrating such interventions into complex critical care pathways.

The CR protocol employed in this study was explicitly designed to address the unique physiological and developmental needs of infants. It integrated three core, synergistic components: a structured exercise intervention, dedicated nutritional support, and systematic developmental care. The respiratory rehabilitation component utilized passive techniques, such as prolonged exhalation and thoracic compression, to address the characteristic tachypnea and limited tidal volume regulation of infants. When combined with targeted airway clearance and, in select cases, external diaphragm pacing, these measures aimed to ameliorate postoperative respiratory dysfunction and diaphragmatic impairment.

Meanwhile, given the substantial developmental heterogeneity within the first year of life, we incorporated strategies to ensure exercise interventions were both safe and appropriate. All infants first underwent assessment using the PDMS-2 to inform individualized program design. Interventions were tailored to developmental stage, age-appropriate milestones, and clinical stability (e.g., passive mobilization for younger infants vs. guided active play for older infants). Furthermore, training intensity was dynamically adjusted based on physiological tolerance. This individualized and adaptive approach was designed to prevent ICU-acquired weakness and promote the sequential achievement of neuromotor milestones. Our integrated model aligns with and extends recent frameworks outlining core components of pediatric cardiac rehabilitation (22) by providing concrete, operationalized strategies for infant-specific adaptation within the ICU setting and by elevating developmental care from a peripheral adjunct to a fundamental pillar of the rehabilitation program.

Nutritional support and developmental care functioned not as isolated elements but as essential, synergistic modules within the holistic rehabilitation protocol. Guided by the principles of “early initiation, enteral priority, and individualized adjustment,” our nutritional strategy encompassed early risk stratification, phase-specific goal setting, and a stepwise transition from parenteral to enteral nutrition. Tailored interventions addressed challenges like swallowing dysfunction and feeding intolerance. The successful transition of all infants to exclusive enteral nutrition provided the crucial metabolic substrate for growth and functional recovery. Concurrently, developmental care measures—focused on environmental modulation, therapeutic positioning, and systematic stress reduction—were implemented in alignment with established neuroprotective recommendations for this population (23). Together, these modules created a comprehensive, rehabilitative milieu that actively supported recovery across multiple domains, exemplifying a multidimensional rehabilitation approach.

The observed recovery trajectories in our cohort progressed across respiratory, feeding, motor, and hemodynamic domains and did not follow a strictly linear pattern. Improvements in vasoactive-inotropic support requirements, respiratory effort, and feeding tolerance emerged in tandem with gains in clinical stability, highlighting the multidimensional and interdependent nature of recovery in critically ill infants. These trajectories likely reflect a complex interaction between innate postoperative physiological stabilization and the facilitative effects of a developmentally guided rehabilitation program. However, elucidating the specific contributions of rehabilitation requires further investigation through larger, controlled studies.

This study has several limitations. As a single-center retrospective case series with a small sample size, its findings are primarily descriptive and hypothesis-generating. The absence of a control group and lack of longitudinal neurodevelopmental follow-up limits causal interpretation. The transfer of two infants to other institutions prior to hospital discharge also resulted in incomplete outcome data for those individuals. Furthermore, while our protocol individualized interventions based on developmental stage, the small sample size precluded formal analysis of how age or specific milestones influenced outcomes. Future studies should incorporate prospective designs with larger cohorts, standardized motor and neurodevelopment assessments, and long-term follow-up into childhood to assess the durability of functional improvements and quality-of-life outcomes.

In conclusion, this study provides preliminary evidence that an early, individualized CR program, tailored to the unique physiological and developmental needs of infants, is both feasible and safe during the postoperative ICU period following complex CHD surgery. The implementation of a multidimensional rehabilitation strategy—integrating structured exercise, dedicated nutritional support, and systematic developmental care—was associated with multi-domain functional recovery during a critical developmental window. These findings support the concept of integrating rehabilitative principles into early postoperative critical care for this vulnerable population. Further research is warranted to rigorously evaluate the effectiveness of such protocols in larger cohorts and to explore implementation strategies for broader clinical adoption once efficacy is established.

## Data Availability

The original contributions presented in the study are included in the article/Supplementary Material, further inquiries can be directed to the corresponding author.
